# The association of CGG repeat length and AGG interruption patterns on FMR1 alleles with female infertility

**DOI:** 10.3389/fendo.2025.1609471

**Published:** 2025-06-17

**Authors:** Zhou Xuanyou, Xu Naixin, Cao Xianling, Shi Weihui, Li Shuyuan, Chen Songchang, Xu Chenming

**Affiliations:** ^1^ Obstetrics and Gynecology Hospital, Institute of Reproduction and Development, Fudan University, Shanghai, China; ^2^ Departments of Obstetrics and Gynecology, Peking Union Medical College Hospital, Chinese Academy of Medical Sciences and Peking Union Medical College, Beijing, China; ^3^ Research Units of Embryo Original Diseases, Chinese Academy of Medical Sciences, Shanghai, China; ^4^ International Peace Maternity and Child Health Hospital, School of Medicine, Shanghai Jiao Tong University, Shanghai, China

**Keywords:** allele 1, FMR1 gene, female fecundity, AGG interruption, fragile X screening

## Abstract

**Background:**

The aim of the present study was to investigate whether the CGG repeat length and AGG interruption patterns on the FMR1 gene affect female fecundity.

**Methods:**

A total of 266 infertile patients and 276 fertile controls were included in the study. All participants received FMR1 testing using triplet repeat primed PCR and capillary electrophoresis. The allele with the smaller number of CGG repeats was defined as "allele 1", and the allele with the larger number of CGG repeats was defined as "allele 2".

**Results:**

The mean number of CGG repeat length at allele 2 in the secondary infertility group was higher than that in the control group (33.1 ± 6.7 vs 30.9 ± 3.3, Bonferroni corrected p=0.003). The proportion of 35–44 CGG repeat at both FMR1 alleles showed a higher trend in the secondary infertility group as compared to the control group after adjusting for age, education, smoking status, cohort and the CGG repeats of the other FMR1 allele (aOR=7.812, 95% CI 0.884-69.001; p=0.064 for allele 1; aOR=3.657, 95% CI 2.193-6.098; p<0.001 for allele 2, respectively). Lower AMH levels were associated with increased CGG repeat length at allele 1 in infertile patients (Adjusted R^2^ = 0.178, p=0.003) after adjusting for age, education, smoking status, infertility type and the CGG repeats of FMR1 allele 2. However, no significant correlation was found between the number of CGG repeats at allele 2 and AMH levels (Adjusted R^2^ = 0.150, p=0.086). Although the difference was not statistically significant, there was a higher proportion of 3 AGG interruptions at both alleles in the secondary infertility group as compared to the control group (6.1% vs 0%, p=0.146 for allele 1, 30.6% vs 11.3%, p=0.099 for allele 2). Patients with 35–44 CGG repeat length showed a higher carrier rate of 3 AGG interruptions at both alleles (p<0.001 for both).

**Conclusions:**

Overall, the high normal sized (35–44 CGG) repeat length at both FMR1 alleles may serve a promoting role in the development of secondary infertility in Asian women. In addition, the CGG repeat length at allele 1 appears to have a mild correlation with AMH levels in infertile patients.

## 1 Introduction

Infertility is defined as an inability to achieve pregnancy after 12 months of unprotected intercourse (1). With the growing global trend for aging population and low fertility rates, infertility has garnered extensive attention in recent years. According to statistics, infertility is estimated to affect as many as 186 million people worldwide (2), accounting for ~15% of couples of childbearing age (3, 4). The incidence and prevalence of infertility vary from country to country, but the overall prevalence has been continuously rising in all countries. The reported rates of infertility are 10-15% in America (5, 6), 15% in Denmark (7), 11.9-17.6% in China (8, 9), 13-20% in Iran (10) and 13.5% in Korea (11). It is estimated that approximately half of infertility cases are attributed to genetic factors, although the underlying causes remain currently unidentified and poorly characterized for most patients (12). Several studies have reported the association between the development of female infertility and genetic polymorphisms (13–15), which indicates that genetic factors may have a substantial role in the development of infertility. As to idiopathic infertility, although its etiology remains elusive, a growing body of evidence indicates a key role of the genetic component involved in the development and progression of the disease. A recent article in the New England Journal of Medicine reported that approximately 17% of the women with idiopathic infertility has pathogenic or likely pathogenic variants for genetic diseases (16).

The fragile X mental retardation gene (FMR1) is localized on chromosome X (Xq27.3), encoding for the FMR1 protein (FMRP), and contains a trinucleotide (CGG) repeat element in the 5'untranslated region (17, 18). A premutation (55–200 CGG trinucleotide repeats in 5' UTR of FMR1 gene) leads to over-representation of FMR1 transcription, aberrant translation products and decline in ovarian function. Premutation (PM) carriers with the above symptoms can be diagnosed as Fragile X primary ovarian insufficiency (FXPOI) (19, 20).

FXPOI is the most commonly known genetic cause for 46, XX POI (21), which is closely related to impaired fecundity. As a consequence, research on the FMR1 gene has extended to other endpoints of reproductive health, including infertility and ovarian reserve (22–27). One study from Latvia reported that the FMR1 gene high-normal alleles are associated with secondary infertility (27), while another study from Switzerland failed to find the association between FMR1 CGG repeat length expansions and infertility (28). Gleicher et al. noted that there is a mild shift toward higher CGG counts at both FMR1 alleles in infertile women in the American population (29). In addition to repeat numbers, the stability of the CGG repeat sequence is also affected by AGG interruptions within the repeat region (30). Since prior studies have shown that AGG interruptions alter the stability of non-canonical structures formed by pathogenic CGG repeats (31, 32), it is possible that AGG interruptions may also play protective roles in fragile X-associated disorders. Recently, Quilichini J et al. have suggested that more than 2 AGG interruptions may be associated with the development of POI, which further confirms the importance of AGG interruptions as a genotypic characteristic for female fecundity (22). The association between FMR1 allele and infertility has been preliminarily explored in the European and American population, however, the relationship between the pattern of AGG interruptions and infertility, especially in Asian population, has not yet been elucidated. The aims of the study, therefore, were to investigate: 1) the relationship of CGG repeat length and AGG interruption patterns at both FMR1 alleles with primary/secondary infertility in the Asian population and 2) whether FMR1 alleles can affect hormone levels in patients with primary/secondary infertility.

## 2 Materials and methods

### 2.1 Patients

A total of 266 patients with primary/secondary infertility and 276 healthy women who had a childbearing history were enrolled in the case-control study. All patients were clinically diagnosed with primary/secondary infertility between July 2019 and July 2023 in the Reproductive Genetic Centre of Obstetrics and Gynecology Hospital affiliated Fudan University (cohort 1) and International Peace Maternal and Child Health Hospital (IPMCH) of Shanghai Jiao Tong University (cohort 2). The inclusion criteria were female infertility: women of reproductive age who failed to achieve pregnancy for more than 1 year regular with unprotected sexual intercourse. Primary infertility (PI) is defined as the state of never achieve a pregnancy while patients who had been pregnant at least once in their lives before were classified in the secondary infertility (SI) group. Non-Chinese patients and patients with definite pathogenic factors, including recurrent spontaneous abortion (RSA), intrauterine adhesion, untreated hydrosalpinx, history of gynecological surgery, malformations in the female genital tract and abnormal karyotype were excluded. To eliminate the effect of male-infertility factors, patients whose husbands were diagnosed with severe oligospermia, asthenospermia, and teratozoospermia were also excluded.

Reproductively active females without history of fertility treatment were selected as the control group. The inclusion criteria were women who had undergone natural conception, no history of *in vitro* fertilization and embryo transfer (IVF-ET), no history of severe systemic diseases, no use of hormonal medications in the previous 3 months, no history of any gynecological endocrine diseases.

In cohort 1, 136 patients and 97 controls were collected from Obstetrics and Gynecology Hospital affiliated Fudan University and the rest 130 patients and 179 controls from IPMCH were in cohort 2. The study was approved by the research ethics committee of the above two hospitals. All patients were informed of details of the procedure and signed the informed consent agreement.

### 2.2 Laboratory testing

The genomic DNA was extracted from peripheral blood samples using the DNA extraction kit (QIAGEN, Germany). The *FMR1* genotype was tested by triplet repeat primed PCR (TP-PCR) and capillary electrophoresis using the protocol previously described (33, 34). FMR1 testing assay in Cohort 1 was performed by the BGI-Huada Clinical Examination Center (Shenzhen, China) following its operating standards while FMR1 CGG repeats test kit (Fluorescence PCR-Capillary Electrophoresis) of Shanghai Pinnacles Medical Technology Co., Ltd. was used in Cohort 2. The allele with the smaller number of CGG repeats was defined as "allele 1", and the allele with the larger number of CGG repeats was defined as "allele 2"^3^. Participants were divided into four groups based on allele 1 or allele 2 (<35, 35-44, 45–54 and 55–200 CGG repeats), as described previously (35, 36). The range of < 35 and 35–44 CGG repeats was defined as normal and high normal, 45–54 CGG repeats were defined as intermediate or grey zone mutation, and 55–200 CGG repeats were defined as premutation.

The TP-PCR strategy employs a chimeric primer containing five consecutive CGG repeats in combination with conventional forward and reverse primers, to enable amplification of the polymorphic CGG tract within the FMR1 gene. The distribution of AGG interruptions was determined by the analysis of the signal loss of five CGG peaks and later recovery of the signal intensity on the electropherograms, as described before (33). Normally, FMR1 alleles with normal size were found to be interrupted with 0, 1, 2, or 3 AGG interruptions.

Normal hormone levels on menstrual days 3–5, including follicle-stimulating hormone (FSH) and anti-Müllerian hormone (AMH), were measured using a chemiluminescence immunoassay (Roche^®^, Switzerland) according to standardized operation process in the hospitals.

### 2.3 Study design

All patients were categorized into three groups: PI, SI and control group. Demographic information and genotype data for participants in these three groups were collected from the hospital electronic medical record system. We first compared the distribution of CGG repeat length at FMR1 allele 1 or 2 among groups. Demographic variables, cohort information, and the other allele were included in the generalized linear model (GLM) and logistic regression model. Subsequently, the relationship between the repeat length at FMR1 alleles and reproductive hormone levels was investigated in the infertile population. Serum FSH and AMH levels were further measured across allele-stratified subgroups (<35 and 35–44 CGG repeats). In addition, we explored the pattern of AGG interruption at both FMR1 alleles among PI, SI and control groups.

### 2.4 Statistical analysis

Data were presented as the means ± SD for continuous variables and frequencies (n, %) for categorical variables. Significant differences in continuous variables between the groups were evaluated by Mann-Whitney U test or Kruskal-Wallis test. The comparison of categorical variables between groups was performed using the chi-square test and Fisher's exact chi-square test. Multivariate analysis with GLM and multivariate logistic regression was performed to adjust for relevant confounding factors. The relationships between FMR1 repeat length and reproductive hormone levels (AMH and FSH) were assessed by linear regression analysis and the results were graphically illustrated using scatter plots. Statistical analysis were performed using SPSS 27.0 for Windows (IBM Corp, USA) and GraphPad Prism (version 9) software (GraphPad Inc., USA) and differences were considered statistically significant at p < 0.05.

## 3 Results

A total of 266 patients and 276 controls undergoing *FMR1* testing participated in our study. The basic characteristics and FMR1 allelic data of patients in the PI, SI and control groups were presented in **Table 1** and **Supplementary Figure 1**. The most prevalent CGG repeat length at allele 1 was 29 CGG in all groups (48.6% in the PI group, 46.8% in the SI group and 47.1% in the control group). The mean number of CGG repeat length at allele 1 was 28.7 ± 2.3 in the PI group, 29.1 ± 3.1 in the SI group and 28.6 ± 2.5 in control patients, the values were not significantly different among the three groups (p=0.478). As for allele 2, the most prevalent CGG repeat length was 29 CGG (30.6%) in the SI group and 30 CGG in the other two groups (33.1% for PI group and 34.1% for control group). The mean number of CGG repeat length at allele 2 in the PI (31.8 ± 3.8) and SI (33.1 ± 6.7) group was higher than that in the control group (30.9 ± 3.3), however, only the difference between SI and control group was statistically significant (Bonferroni corrected p=0.003). Only one PM allele (87 CGG repeat length) was found in the SI group, while it was not observed among the control patients. Age, education level and smoking status showed no significant differences among the three groups (p=0.076, p=0.699, p=0.546, respectively). In addition, there was no significant difference in the etiology distribution between the PI and SI groups (p=0.950).

**Table 1:** Basic characteristics of patients in PI,SI and control groups.

Variables	PI N=142	SI N=124	Control N=276	P
Age, y	32.8 ± 4.4	33.6 ± 4.3	32.4± 3.1	0.076
Education, n (%)				0.699
Senior high school degree or less	16 (11.3%)	19 (15.3%)	45 (16.3%)	
college degree	113 (79.6%)	93 (75.0%)	209 (75.7%)	
post-graduate degree	13 (9.2%)	12 (9.7%)	22 (8.0%)	
Smoking status, n (%)				0.546
Y	4 (2.8%)	1 (0.8%)	7 (2.5%)	
N	138 (97.2%)	123 (99.2%)	269 (97.5%)	
Infertility etiology, n (%)				0.950^a^
Anovulation	13 (9.2%)	11 (8.9%)		
Primary ovarian insufficiency	4 (2.8%)	5 (4.0%)		
Endometriosis	2 (1.4%)	1 (0.8%)		
Unexplained	123 (86.6%)	107 (86.3%)		
FMR1 Allele1				0.478
FMR1 Allele1, mean ± SD	28.7 ± 2.3	29.1 ± 3.1	28.6 ± 2.5	
FMR1 Allele1, the most common allele	29 (69, 48.6%)	29 (58, 46.8%)	29 (130, 47.1%)	
FMR1 Allele1, Range	18-37	11-40	11-35	
FMR1 Allele2				0.003*^b^
FMR1 Allele2, mean ± SD	31.8 ± 3.8	33.1 ± 6.7	30.9 ± 3.3	
FMR1 Allele2, the most common allele	30 (47, 33.1%)	29 (38, 30.6%)	30 (94, 34.1%)	
FMR1 Allele2, Range	24-48	23-87	11-45	

Continuous variables were calculated by Kruskal-Wallis test, categorical variables were calculated by chi-squared test; p value for comparisons among PI,SI and control groups; ^a^ represent p value for comparison between PI and SI group. ^b^ represent Bonferroni corrected p value for comparison between SI and control group. PI, Primary Infertility; SI, Secondary Infertility; *p<0.05.

In order to investigate the association of the repeats on FMR1 gene and infertility, linear regression analysis with GLM model for the CGG repeats on both alleles were performed (**Table 2**). The associations of the CGG repeat length at allele 1 with PI or SI remained nonsignificant after adjusting for potential confounding factors including age, education, smoking status, cohort and the CGG repeat length at FMR1 allele 2 (aOR 1.204, 95% CI 0.716- 2.027, p=0.484 for PI; aOR 1.216, 95% CI 0.706- 2.097, p=0.481 for SI). In addition, the number of CGG repeats at allele 2 were still associated with SI when adjusting for age, education, smoking status, cohort and the CGG repeat length at FMR1 allele 1 (aOR 7.366, 95% CI 2.983-18.188, p<0.001).

**Table 2:** Results of GLM models for the association of CGG repeats at FMR1 alleles with primary Infertility and secondary Infertility.

FMR1 CGG repeats	PI		SI	
	aOR (95% CI) P		aOR (95% CI) P	
FMR1 Allele 1^a^	1.204 (0.716- 2.027)	0.484	1.216 (0.706- 2.097)	0.481
FMR1 Allele 2^b^	2.061 (0.858-4.950)	0.106	7.366 (2.983-18.188)	<0.001*

a adjusted for age, education, smoking status, cohort and CGG repeats of FMR1 allele 2.

b adjusted for age, education, smoking status cohort and CGG repeats of FMR1 allele 1.

*p<0.05; PI, Primary Infertility; SI, Secondary Infertility;aOR, adjusted odds ratio; CI, confidence interval.

**Figure 1:**
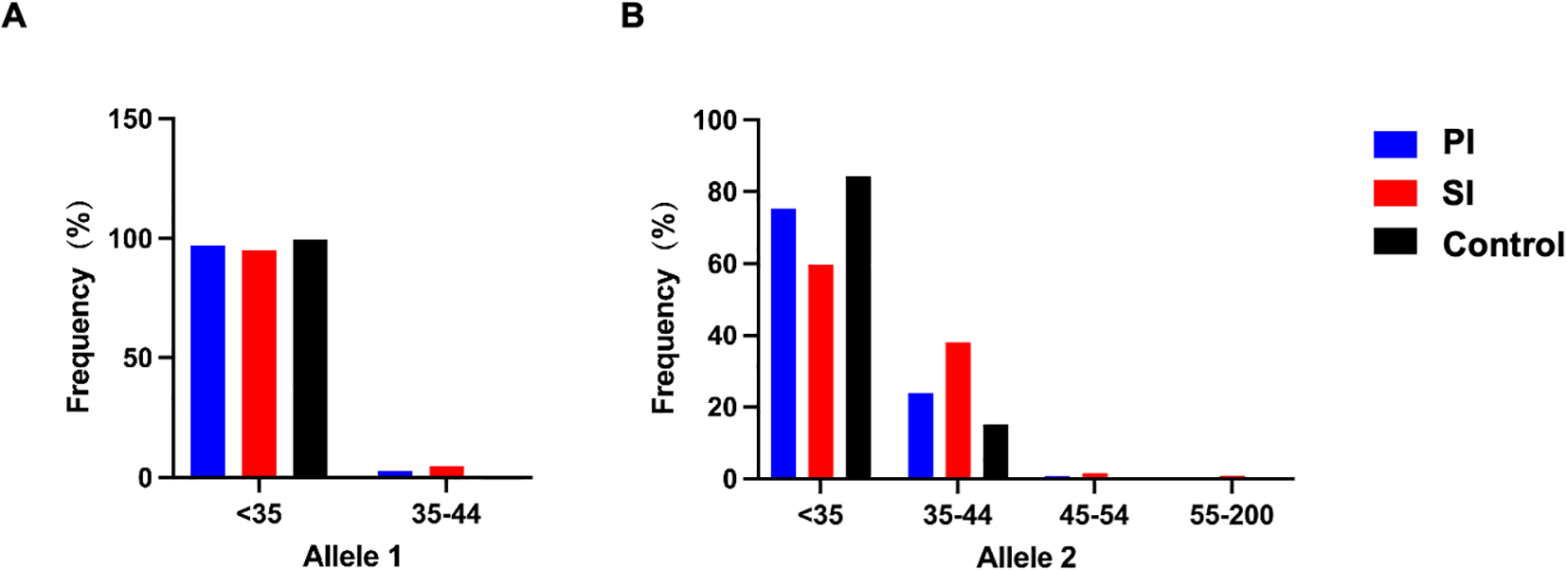
FMR1 allelic frequency with categories of FMR1 allele 1 **(A)** and allele 2 **(B)** in the PI (blue), SI (red) and control group (black). The FMR1 CGG repeat lengths were categorized as follows: <35 CGG repeats: normal range, 35–44 CGG repeats: high normal range, 45–54 CGG repeats: intermediate or grey zone mutation, 55–200 CGG repeats: premutation. PI, Primary Infertility; SI, Secondary Infertility.

To further clarify the specific range of repeat numbers which is associated with infertility, participants were divided into four consecutive FMR1 groups according to the repeat lengths at both alleles. The results were shown in **Table 3** and **Figure 1**. A different distribution pattern was observed among PI, SI and control groups in both alleles (p=0.005, p<0.001, respectively). For allele 1, when compared to controls, the proportion of patients with 35–44 CGG repeat at FMR1 allele 1 was higher in the PI group and SI group (2.8% vs 0.4%, 4.8% vs 0.4%, respectively; **Figure 1A**). For allele 2, there was also a higher proportion of patients with 35–44 repeats in the PI group and SI group than in the control group (23.9% vs 15.2%, 37.9% vs 15.2%, respectively; **Figure 1B**). The results of multivariate logistic regression were shown in **Table 4**. After adjusting for age, education, smoking status, cohort and the CGG repeat length at FMR1 allele 2, the CGG repeat length at FMR1 allele 1 was not associated with PI (p=0.108), while the association between the CGG repeat length at FMR1 allele 1 and SI showed a trend toward significance (p=0.064). For allele 2, the differences in the proportion of 35–44 repeats between groups remained significant when adjusting for age, education, smoking status, cohort and the CGG repeats at FMR1 allele 1 (aOR=1.751, 95% CI 1.028- 2.985; p=0.039 for PI; aOR=3.657, 95% CI 2.193- 6.098; p<0.001 for SI, respectively). Furthermore, the proportion of the other sized CGG repeats did not differ between groups after adjusting for potential confounding factors (p=ns for all).

**Table 3:** Detailed distribution of CGG repeats at FMR1 allele 1 or allele 2 in infertile patients and controls.

FMR1 Categories	PI N=142	SI N=124	Control N=276	P
Allele 1				0.005*
<35	138 (97.2%)	118 (95.2%)	275 (99.6%)	
35-44	4 (2.8%)	6 (4.8%)	1 (0.4%)	
45-54	–	–	–	
55-200	–	–	–	
Allele 2				<0.001*
<35	107 (75.4%)	74 (59.7%)	233 (84.4%)	
35-44	34 (23.9%)	47 (37.9%)	42 (15.2%)	
45-54	1 (0.7%)	2 (1.6%)	1 (0.4%)	
55-200	0 (0.0%)	1 (0.8%)	0 (0.0%)	

Fisher's exact test was used for analysis; p values represent significance levels in the PI, SI group and control group; *p<0.05; PI, Primary Infertility; SI, Secondary Infertility.

**Table 4:** Results of multivariate logistic regression models with categories of FMR1 CGG repeat numbers for infertile patients.

FMR1 Categories	PI		SI	
	aOR (95% CI) P		aOR (95% CI) P	
FMR1 Allele 1^a^				
<35	ref	ref	ref	ref
35-44	6.346 (0.667- 60.355)	0.108	7.812 (0.884- 69.001)	0.064
45-54	–	–	–	
55-200	–	–	–	
FMR1 Allele 2^b^				
<35	ref	ref	ref	ref
35-44	1.751 (1.028- 2.985)	0.039*	3.657 (2.193- 6.098)	<0.001*
45-54	2.553 (0.148- 44.147)	0.519	7.083(0.597- 83.994)	0.121
55-200	–	–	–	0.998

a adjusted for age, education, smoking status, cohort and CGG repeats of FMR1 allele 2.

b adjusted for age, education, smoking status cohort and CGG repeats of FMR1 allele 1.

*p<0.05; PI, Primary Infertility; SI, Secondary Infertility;aOR, adjusted odds ratio; CI, confidence interval.

**Figure 2:**
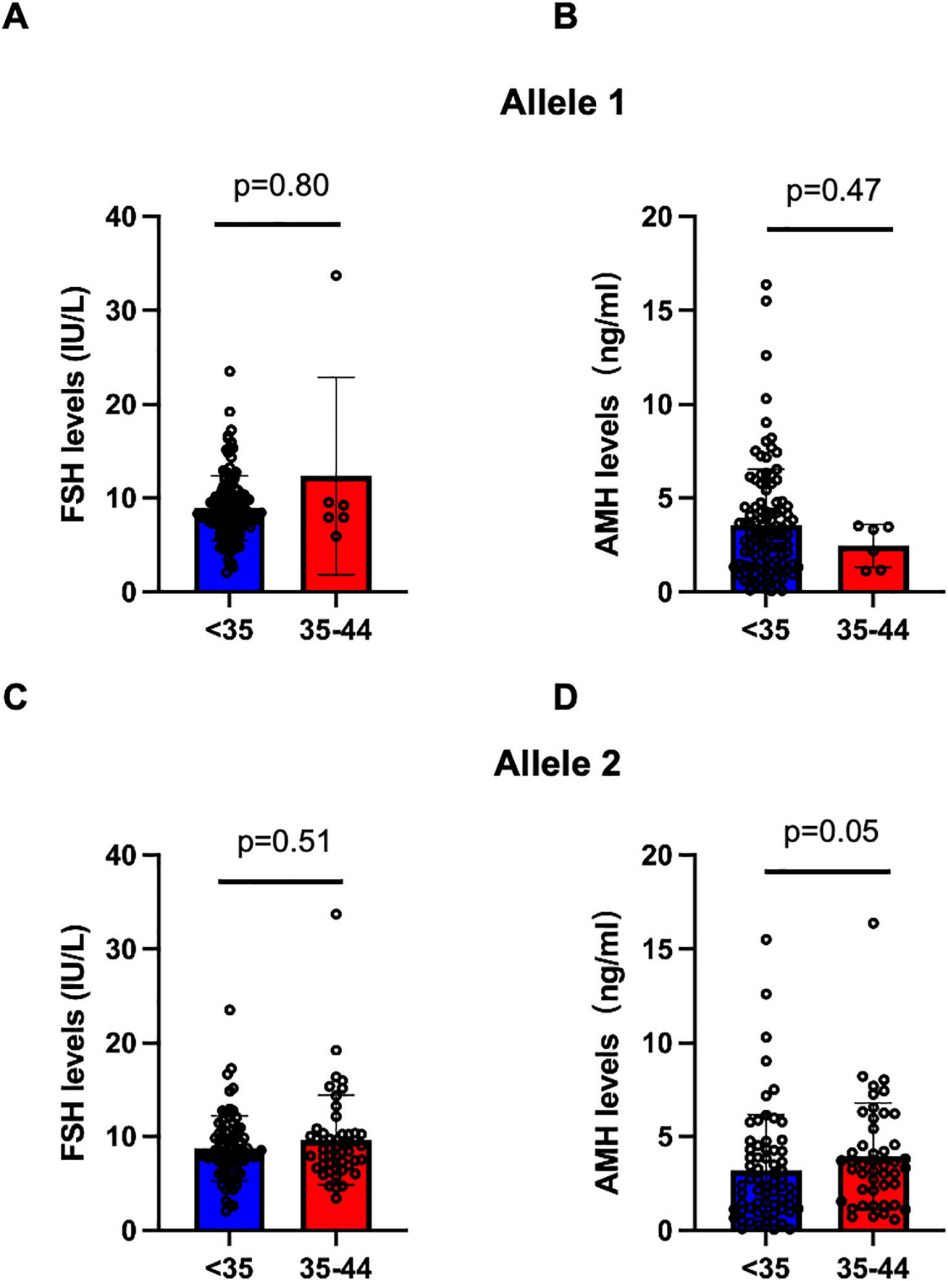
Comparision of serum FSH and AMH levels in the <35 (blue) and 35-44 (red) CGG subgroups at FMR1 allele 1 **(A, B)** and allele 2 **(C, D)**. Bar graphs are presented as means ± SD, each dot represents one patient.

**Figure 3:**
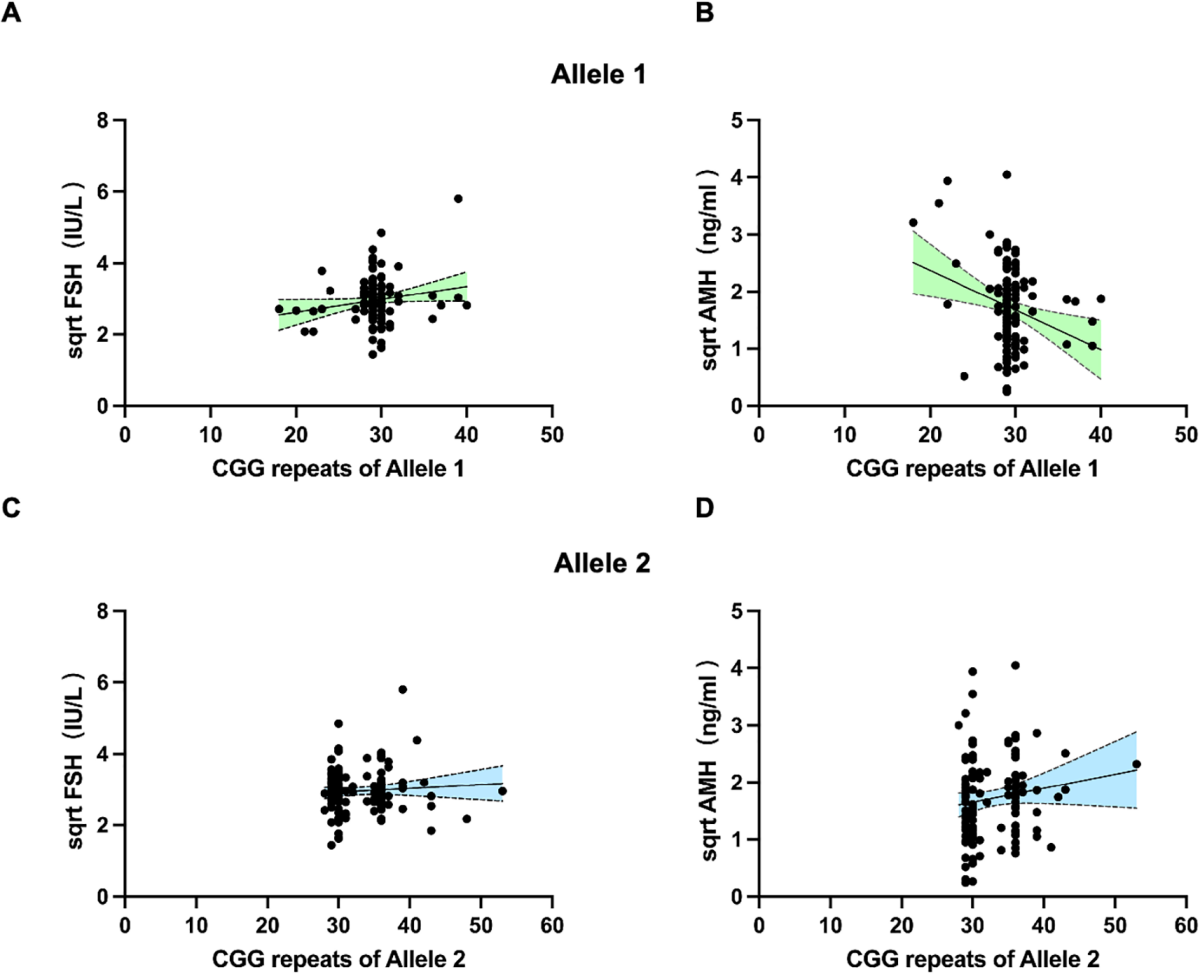
Scatter plots illustrating the association between FMR1 CGG repeats of allele 1 **(A, B)** and allele 2 **(C, D)** with FSH and AMH levels. Dotted lines (grey) represent the 95% confidence intervals.

As we found the association between 35–44 CGG repeat length at both FMR1 alleles and SI in the above analysis, we further investigated the serum FSH and AMH levels between <35 and 35–44 CGG repeats subgroups (**Figure 2**). The levels of serum FSH did not differ significantly between groups (p=0.80 for allele 1, p=0.51 for allele 2, respectively; **Figures 2A, C**). The mean value of AMH levels in 35–44 CGG repeats subgroup at allele 1 was lower than that in <35 CGG repeats subgroup, although the difference was not statistically significant (p=0.47, **Figure 2B**). A higher AMH levels was observed in 35–44 CGG repeats subgroup at allele 2 as compared with <35 CGG repeats subgroup, similarly, there was no statistically significant difference (p=0.05, **Figure 2D**). Subsequently, we investigated the relationship between FMR1 alleles and reproductive hormone levels in patients with infertility (**Figure 3**). After adjusting for confounding factors including age, education, smoking status, infertility type and the CGG repeats of the other FMR1 allele, there was no significant difference in FSH levels with the change of CGG repeat length at FMR1 allele 1 or 2 (p=0.110, p=0.429, respectively; **Figures 3A, C**). Decreased AMH levels were associated with an increase in CGG repeat at allele 1 after adjusting for confounding factors including age, education, smoking status, infertility type and the CGG repeats of FMR1 allele 2 (Adjusted R^2^ = 0.178, p=0.003; **Figure 3B**). However, no significant correlation was found between the number of CGG repeats at allele 2 and AMH levels (Adjusted R^2^ = 0.150, p=0.086; **Figure 3D**).

**Figure 4:**
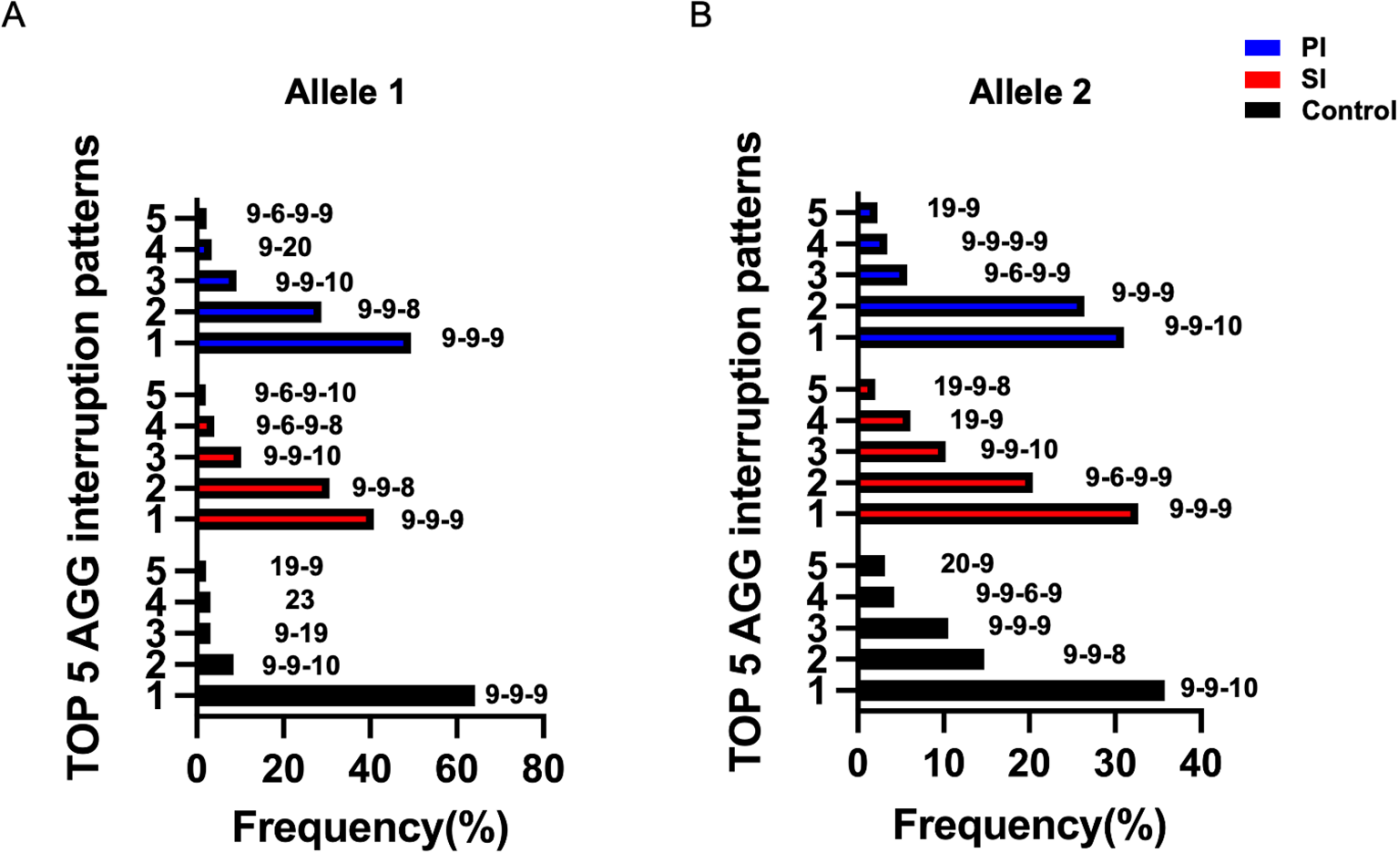
The top five highest-frequency AGG interruption patterns at FMR1 allele 1 **(A)** and allele 2 **(B)** in the PI (blue), SI (red) and control group (black); PI, Primary Infertility; SI, Secondary Infertility.

**Figure 5:**
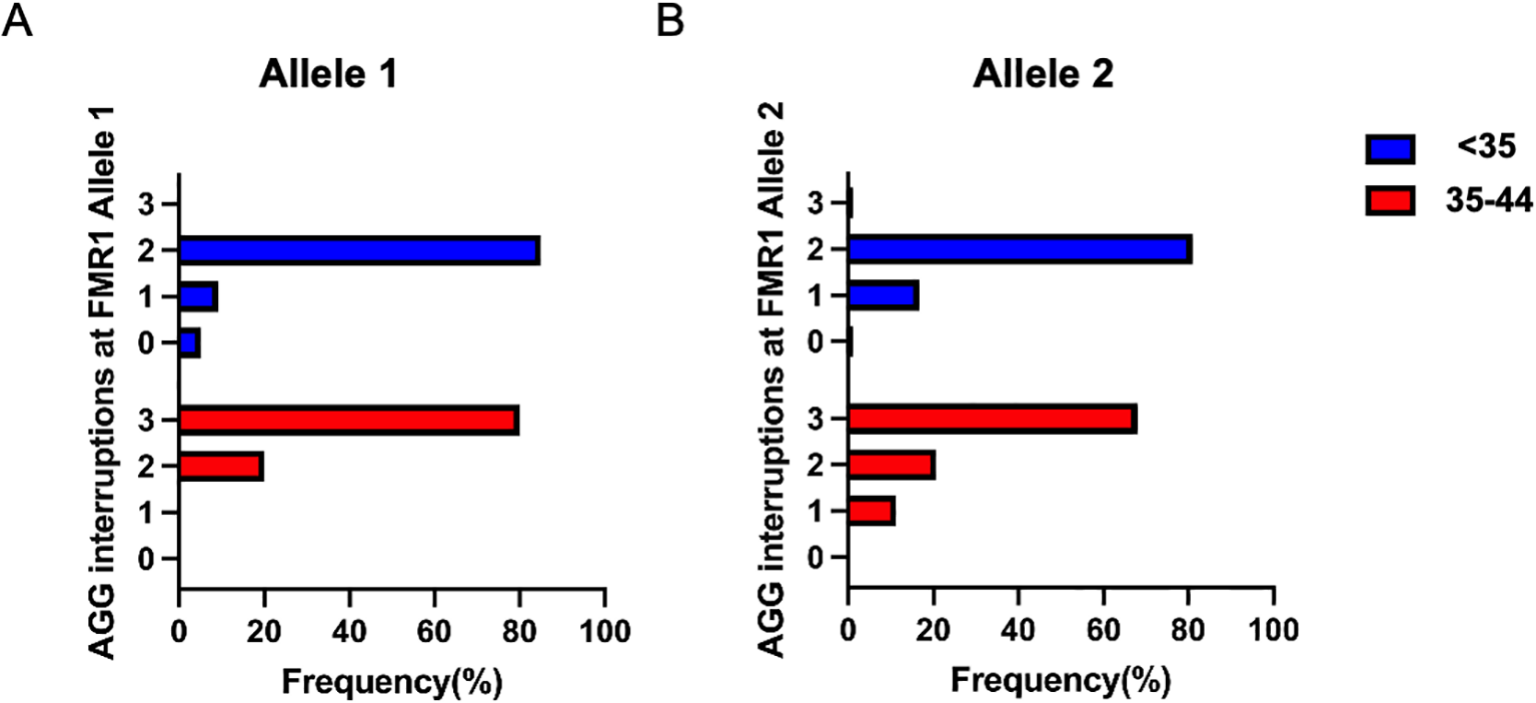
The number of AGG interruptions in the <35 (blue) and 35-44 (red) CGG subgroups at FMR1 allele 1 **(A)** and allele 2 **(B)**.

The pattern of AGG interruption on the FMR1 alleles was also investigated in the study. The top five highest-frequency AGG interruption patterns in the PI, SI and control group were presented in **Figure 4**. For allele 1, the top three ranked patterns were 9-A-9-A-9, 9-A-9-A-8 and 9-A-9-A-10 in the PI and SI group, 9-A-9-A-9, 9-A-9-A-10 and 9-A-19 in the control group (**Figure 4A**). Patients in the PI and SI groups showed a 3 AGG pattern (9-A-6-A-9-A-9, 9-A-6-A-9-A-8) for the fourth or fifth ranked AGG interruption pattern while controls carried a continuous stretch of 23 without any AGG interruptions and 19–9 CGG repeats for the same ranks. For allele 2, the top 3 AGG interruption patterns were 9-A-9-A-10, 9-A-9-A-9, 9-A-6-A-9-A-9 in the PI group, 9-A-9-A-9, 9-A-6-A-9-A-9 and 9-A-9-A-10 in the SI group and 9-A-9-A-10, 9-A-9-A-8, 9-A-9-A-9 in the control group (**Figure 4B**). To summarize, the pattern of 3 AGG interruptions (9-A-6-A-9-A-8, 9-A-6-A-9-A-10, 9-A-6-A-9-A-9) appeared earlier in the PI and SI group than in the control group, which indicated that patients with 3 AGG pattern accounted for a higher proportion in the PI and SI group rather than in the control group. The results were corroborated by further analysis of the number of AGG interruptions among different groups (**Supplementary Table 1**). Although the difference was not statistically significant, there was a higher proportion of 3 AGG interruptions at allele 2 in the SI group as compared to the control group (30.6% vs 11.3%, p=0.099). The proportion of 3 AGG interruptions at allele 1 in the SI group was also higher than that in control group (6.1% vs 0%, p=0.146). We then compared the number of AGG interruptions at allele 1 and allele 2 between patients with <35 and 35–44 CGG repeat length (**Figure 5**). Patients with 35–44 CGG repeat length showed a higher carrier rate of 3 AGG interruptions at both allele 1 and allele 2 (p<0.001 for both).

## 4 Discussion

Primary ovarian insufficiency (POI), or primary ovarian failure (POF), is now more recognized as a pathological state of ovarian hypofunction, menstrual irregularity, infrequent or absent ovulation. Female fecundity can be impaired by any condition that affects ovulation rate, ultimately leading to infertility (37). FMR1 gene is the most prominent candidate gene of POI, as a consequence, it has been postulated that a relationship exists between FMR1 gene and female infertility (38). However, there is relatively little published studies on the effects of FMR1 CGG repeat size on female infertility, especially in the Asian population. In the study, we firstly analyzed the relationship of FMR1 CGG repeats with PI or SI, we found a higher number of CGG repeats at allele 2 were associated with SI. Patients in the SI group seem to have a higher repeat size (35–44 CGG) at both alleles. Our results first confirmed the relation between higher FMR1 repeat number and SI in the Asian population. Previously, only one publication based on the European population reported the relationship of high normal (35–54 CGG) sized FMR1 repeat length with SI (27). Another study conducted by De Geyter et al. reported that the distribution of FMR1 CGG repeat length did not differed between infertile women and control women (28). Differences in study inclusion criteria may explain the inconsistent results between two European population-based studies. In the former study, the inclusion criteria of patients were SI while whole infertile patients, including patients with PI or SI, were involved in the study by De Geyter et al. (28). Though collectively termed as infertility, these two diseases have vastly different characteristics and molecular pathogenesis. Therefore, it was reasonable for the inconsistent findings in the above two studies. In fact, inconsistent findings of the PI and SI group were also observed in our study, which suggest that FMR1 gene may be more closely associated with SI rather than PI. The underlying reason remains unclear, but it could be attributed to the impact of the FMR1 gene on ovarian function occurring during adulthood rather than affecting the follicle pool (39). The mutation of the FMR1 gene may drive premature depletion of follicles, thereby shortening the reproductive lifespan, ultimately leading to secondary infertility. Since it does not affect the primordial follicle pool, it is unlikely to be a direct contributor to primary infertility.

It is generally believed that the pathogenic mechanism of CGG repeat expansions may be associated with two pathways: either the detrimental effects arising from the overexpression of RNA containing long CGG repeat sequences, or the toxicity caused by products generated through repeat-associated non-AUG translation. The pathogenic products derived from the FMR1 gene impair female fertility by affecting follicular development, inducing follicular depletion and compromising ovarian reserve. One study has demonstrated that the transcriptional activity of CGG repeats ranging from 41–60 is higher than that of repeats with fewer than 40 CGG repeats, which indicates that repeat sequences which have not yet reached the premutation threshold or are in the vicinity of this threshold may still exert potential toxic effects. Although no direct evidence has confirmed a definitive causal relationship between high normal sized CGG repeat sequence and RNA toxicity or FMRpolyG-associated pathology, several studies have provided substantial evidence supporting the potential reproductive toxicity of the alleles (27, 40, 41). In the study, we speculate that lower reproductive function of patients with 35–44 CGG repeat length may be related to reduced ovarian reserve, however, we failed to find decreased AMH levels or increased FSH levels in infertile patients with 35–44 CGG repeat length at allele 2. Patients with 35–44 CGG repeats at allele 1 seem to have a lower AMH levels as compared to patients with <35 CGG repeats, the difference was not statistically significant possibly because of the small sample size. Our results also suggest a modest negative correlation between AMH levels and CGG repeat length at allele 1. Previous studies suggest that FMR1 repeat length had limited predictive ability for ovarian reserve and most of these findings have been reported in American and European populations (28, 42, 43). Considering the unique role of FMR1 allele 1 in the Asian population (44, 45), we supposed that the CGG repeat length on allele 1 appears to have a stronger correlation with AMH levels than allele 2 in Asian women.

Our study represents the first investigation of the association between the pattern of AGG interruption at the FMR1 alleles and female infertility. The AGG interruption at the FMR1 alleles generally includes the following four patterns: 0, 1, 2, or 3 in the expansion of CGG repeat tracts, which can affect allele instability during transmission. Several studies have reported that there was a trend towards the absence of AGG interruption patterns in patients with POF or RSA as compared to that in control women (44, 46). This led us to ask whether a higher proportion of pure CGG repeat tract will be found in infertile patients. Unexpectedly, we did not observe a dramatic increase of 0-AGG interruption patterns in PI or SI group compared to controls. The SI group showed a higher proportion of 3 AGG interruptions at both alleles than in controls. Patients with 35–44 CGG repeat length also showed a higher carrier rate of 3 AGG interruptions at both alleles. It has been shown that the most common AGG pattern of 3 interruptions is 9-A-6-A-9-A-9 (36 CGG repeats) (47, 48), thus, the findings further confirm the unique associations between 35–44 CGG repeat length and SI.

At present, carriers with high normal sized (35–44 CGG) repeat length receive little attention during genetic counseling practice. Female carriers with high normal sized (35–44 CGG) repeat length should also be informed on the potential risk of subfertility, and family members should be offered fragile-X screening and genetic counselling. Further, with the growing demand for extending the effective reproductive age of females, it is necessary for clinicians to increase the attention towards the fertility distress of females with high normal sized (35–44 CGG) repeat length, so as to timely provide fertility guidance for these carriers.

The major limitations of the current study are as follows. Firstly, this study was conducted as a retrospective case-control study with single-ethnicity participants, which may affect the extrapolation of results. Secondly, the sample size of patients who underwent reproductive hormones testing was small, limited sample size affected the accuracy of the results.

Overall, our study suggests that in the Chinese population, the proportions of patients with 35–44 CGG repeat at both FMR1 alleles were higher in patients with SI when compared with controls. The finding reveals a positive association between high normal sized CGG repeat pattern and secondary infertility, the underlying mechanism merits further investigation. In addition, we also find that the CGG repeat length on FMR1 allele 1 are mildly correlated with serum AMH levels in infertile patients.

## Data Availability

The raw data supporting the conclusions of this article will be made available by the authors, without undue reservation.
