# Supplementary material for: The association of CGG repeat length and AGG interruption patterns on FMR1 alleles with female infertility
Source: Front Endocrinol (Lausanne). 2025 Jun 17;16:1609471. doi: 10.3389/fendo.2025.1609471 (PMC12208826; doi:10.3389/fendo.2025.1609471)
Supplement: Supplementary file 1 [file DataSheet1.docx]

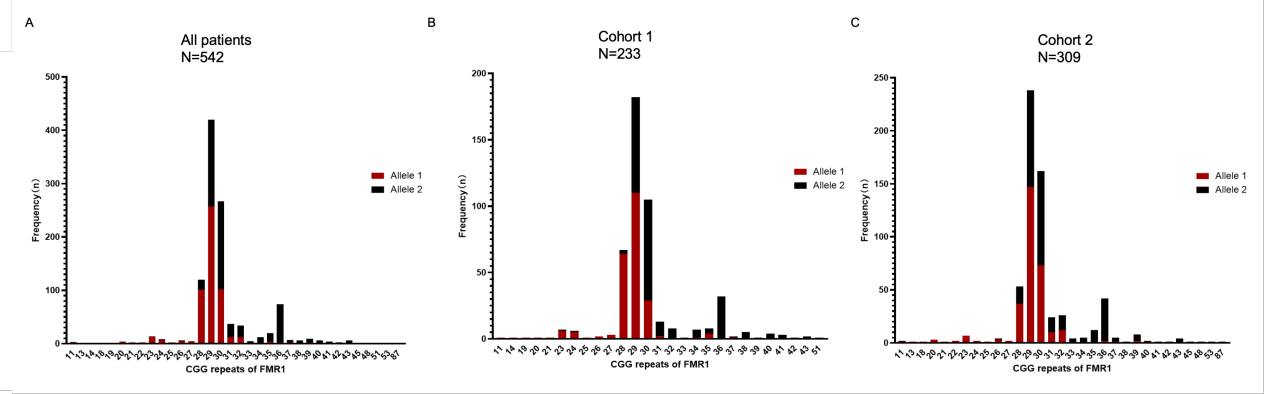


**Supplementary Figure 1** FMR1 allelic frequency of the whole study population in all patients (A), cohort 1 (B) and cohort 2 (C).

**Supplementary Table 1** The number of AGG interruptions in patients with Infertility and controls.

| AGG interruptions on FMR1 gene | PI  N=87 | SI  N=49 | Control  N=97 | P |
| --- | --- | --- | --- | --- |
| **Allele 1** |  |  |  | 0.146 |
| 0 | 3（3.4%） | 1（2%） | 8（8.2%） |  |
| 1 | 6（6.9%） | 5（10.2%） | 10（10.3%） |  |
| 2 | 76（87.4%） | 40（81.6%） | 79（81.4%） |  |
| 3 | 2（2.3%） | 3（6.1%） | 0（0%） |  |
| **Allele 2** |  |  |  | 0.099 |
| 0 | 1（1.1%） | 1（2.0%） | 1（1%） |  |
| 1 | 14（16.1） | 5（10.2%） | 17（17.5%） |  |
| 2 | 60（69.0%） | 28（57.1%） | 68（70.1%） |  |
| 3 | 12（13.8%） | 15（30.6%） | 11（11.3%） |  |

Fisher’s exact test was used for analysis; p values represent significance levels among PI, SI and control group; Abbreviation: PI, Primary Infertility; SI, Secondary Infertility;
